# Ecotoxicological Effects of a Commercial Fenthion-Based Formulation on the Survival, Hatching, and Growth of *Artemia salina* L.: Implications for Risk Assessment in Marine and Estuarine Environments

**DOI:** 10.1021/acsomega.6c06726

**Published:** 2026-10-27

**Authors:** Leonardo Mendes da Silva, Rafael Pereira Ramos, Joice Lima Ferreira, João Lucas Fonseca Landim, Maria Eduarda Magalhães, Larissa Fonseca Andrade-Vieira

**Affiliations:** Departamento de Ecologia e Conservação, 67739 Universidade Federal de Lavras, 37200-900 Lavras, MG, Brazil

## Abstract

This present study evaluated the effects of a commercial formulation based on fenthion on survival, hatching, and growth of *Artemia salina*, and characterized the environmental risk of this insecticide to marine and estuarine ecosystems. The fenthion-based commercial formulation was tested at concentrations ranging from 0.001 up to 731 mg/L using different bioassays: a standard 48-h acute test, a 14-day long-term test, and also two 72-h assays (one for cyst hatching, another for growth inhibition). Obtained EC_50_ values were fully integrated with available literature data to construct a species sensitivity distribution (SSD) curve, which then determined the hazardous concentration for 5% of species (HC_5_), the predicted no-effect concentration (PNEC), and the risk quotient (RQ). In the acute assay, the formulation showed low toxicity, with EC_50_ of 212.36 mg/L at 24 h and 16.64 mg/L at 48 h. In contrast, high toxicity was observed in long-term tests, with EC_50_ of 0.0752 mg/L after 8 days and 0.0052 mg/L after 14 days of exposure. Minor effects were observed on hatching rate (EC_50_ = 101.75 mg/L), whereas more pronounced effects were seen on hatching speed (EC_50_ = 19.88 mg/L) and nauplii growth (EC_10_ = 0.076 mg/L). The HC_5_ from the SSD was 0.000104 mg/L, while PNEC was 0.000020 mg/L. The average RQ was 1.86, indicating high risk. Overall, these results demonstrate the ecotoxicological potential of a fenthion-based commercial formulation for relevant organisms in marine and estuarine ecosystems, reinforcing the importance of regulatory measures and environmental monitoring in areas subject to contamination by this insecticide.

## Graphical abstract

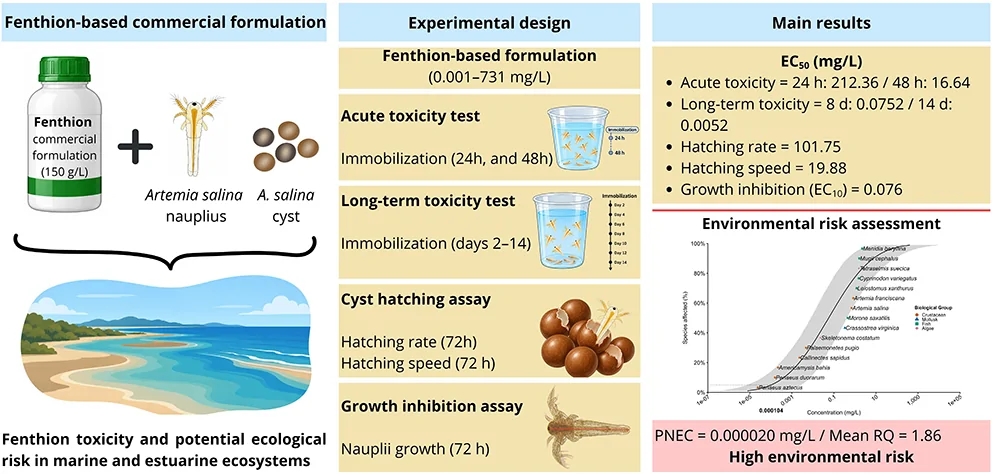

## Introduction

Pesticide use is widely disseminated across multiple productive sectors. At the global scale, the total volume of these compounds reached 3.73 million tons of active ingredients in 2023. Beyond the amount used, international trade reinforces this dissemination: in the same year, the Americas were the region that imported the most pesticides from other locations (1.97 million tons), while Asia led exports (2.4 million tons),^1^ evidencing a robust global flow of formulations and active ingredients. Among pesticides, insecticides are the second most commercialized class worldwide, representing approximately 29.5% of the total.^2^ These compounds are widely used as agricultural inputs to minimize losses caused by arthropod pests in cropping systems,^3^ and, in certain contexts, they are also used in the sanitary management of livestock production to control ectoparasites.^4,5^ Alongside agronomic benefits, recent literature has shown that widespread insecticide use is associated with soil contamination^6^ and water contamination,^7^ in addition to causing impacts on nontarget organisms.^8^

Among insecticides whose ecotoxicological evidence base still presents relevant gaps for a robust risk assessment, fenthion stands out, an organophosphate whose toxicity mechanism is related to cholinesterase inhibition.^9,10^ From a regulatory perspective, this toxicological profile, together with uncertainties in risk assessments, has resulted in formal restrictions: in the European Union, Decision 2004/140/EC determined the noninclusion of fenthion and the withdrawal of authorizations for plant protection products, due to the finding of very high acute risk to birds and insufficient data to ensure environmental safety, including regarding sublethal effects.^11^ In the United States, the EPA issued a cancellation order prohibiting the sale, distribution, and use of the compound after November 30, 2004, in accordance with applicable federal law.^12^ More recently, at the international level, the Rotterdam Convention included ULV fenthion formulations (≥640 g a.i./L) in Annex III, classifying them as severely hazardous pesticide formulations, with entry into force on October 22, 2025,^13^ which reinforces the need for ecotoxicological studies and risk assessments in contexts where the active ingredient remains in use.

Despite early bans in major jurisdictions, the compound remains available in Brazil, both as a standalone ingredient in registered agroveterinary products (license 10.097/2015)^14^ and in formulations associated with other active ingredients.^5^ In the African continent, fenthion is still widely used as an avicide (*Quelea quelea*),^15^ and it is also employed in the control of pests of public health importance, such as malaria and dengue.^16^ In Pakistan, the use of the compound is authorized for insect control in rice and cotton crops.^17^ In China, formulations containing fenthion are registered for “sanitary use,” aimed at controlling fly and mosquito larvae.^18^

Considering its use in distinct contexts, agricultural, livestock, and urban, both fenthion and its metabolites have been frequently detected in aquatic environmental matrices. Xiao et al.,^19^ for example, reported that fenthion was among the three most prevalent pesticides in the East China Sea, with concentrations up to 0.147 ng/L and ecological risk classified as moderate to high. Similarly, Köck-Schulmeyer et al.^20^ detected the metabolite fenthion sulfoxide in seawater samples collected along the Catalonian coast, with a median concentration of 0.69 ng/L, a mean of 1.01 ng/L, and a maximum of 1.67 ng/L. In addition, Peris et al.^21^ identified the transformation products fenthion oxon and fenthion oxon sulfoxide in sediments from the Ebro River Delta, with maximum concentrations of 0.06 and 0.31 ng g^–1^ dw, respectively.

Among the test organisms used in the ecotoxicological assessment of marine and estuarine environments, *A. salina* L. (Crustacea: Anostraca) stands out as a halophilic microcrustacean widely distributed in these ecosystems. Its broad application in bioassays is due, in part, to its ability to produce resistant cysts that can be stored for long periods and hatched under controlled and reproducible conditions.^22^ From an ecological standpoint, *Artemia* sp. can constitute a dominant fraction of the macrozooplankton in saline systems, serving as a consumer of phytoplankton and a food resource for aquatic birds, thereby linking primary producers to higher trophic levels.^23^ Recent studies indicate that the combination of practicality, standardization, and the availability of different life-cycle stages supports its use both in rapid substance screening and in characterizing pesticide risk, since these assays allow measurement of end points such as nauplii survival/mortality,^5,24^ inhibition of cyst hatching,^25,26^ and changes in development/growth (e.g., size, teratogenicity, and progression of larval stages).^26,27^ The possibility of deriving multiple effect measures from distinct biological responses within the same species provides a suitable experimental basis for constructing an Intraspecies End point Sensitivity Distribution (iESD) with *A. salina*.

This approach was originally proposed by Hanson and Solomon^28^ as the Effect Measure Distribution (EMD). Subsequently, Brain et al.^29^ used the term iESD when evaluating *Myriophyllum sibiricum* and *Lemna gibba* exposed to pharmaceutical mixtures. Thus, an iESD probabilistically describes differences in sensitivity among end points within a single species and allows lower percentiles, such as the first and fifth percentiles, to be derived for characterizing intraspecific hazard.^29^ Because these values do not represent protection at the community level, the iESD can be used as an exploratory or preliminary approach when interspecific ecotoxicological data are limited, as may occur in assessments of commercial formulations or poorly studied substances.

Complementing this intraspecific perspective, Species Sensitivity Distributions (SSDs) extend the assessment to the interspecific level by integrating toxicity data from different organisms and allowing the estimation of percentiles, such as the hazardous concentration for 5% (HC_5_) represented in the distribution.^30,31^ In addition to their application in hazard characterization, values derived from SSDs can be used as a basis for deriving reference concentrations in ecological risk assessments. For example, when adequate ecotoxicological data are available, the Government of Canada uses the fifth percentile of the SSD as a basis for deriving a predicted no-effect concentration (PNEC). This concentration can subsequently be compared with environmental exposure levels to calculate the risk quotient (RQ), which is used in ecological risk characterization.^32^ Although iESDs and SSDs share a similar probabilistic framework, they operate at different levels: the iESD describes differences in sensitivity among end points within a single species, whereas the SSD integrates differences among species and supports the derivation of reference values intended for protection at the community level.

In this context, the present study aimed to evaluate the effects of a commercial fenthion-based formulation on the survival of *A. salina* under short- and long-term exposures, as well as on cyst hatching and naupliar growth. The effect concentrations obtained from these bioassays were used to construct an iESD to compare the sensitivity among the evaluated end points and estimate the HC_5_ at the end point level, thereby characterizing variation in sensitivity within *A. salina* that would not be captured by an interspecific SSD. In a second step, the experimental data were combined with ecotoxicological data available in the literature for other marine and estuarine species to construct an SSD for fenthion. From this distribution, the HC_5_ at the community level and the PNEC were estimated. Finally, the PNEC was compared with measured environmental concentrations of fenthion to calculate RQ values and characterize the ecological risk of the compound in marine and estuarine environments.

## Materials and Methods

### Commercial Fenthion-Based Formulation Acquisition

The commercial fenthion-based formulation used in this study was Thion C (Agener União, veterinary division of União Química Farmacêutica Nacional, Brazil; registration No. 10.097/2015), purchased from an agricultural supply store through an electronic transaction. According to the manufacturer’s product information, the formulation contains 150 g/L of fenthion (O,O-dimethyl O-[3-methyl-4-(methylthio)­phenyl] phosphorothioate; CAS 55–38–9), an organophosphate insecticide, and 0.5 g/L of green dye, with vehicle in a quantity sufficient to complete 1 L. The chemical composition of the vehicle is not disclosed by the manufacturer; therefore, the identity and proportion of its individual constituents were not available.

### Test Solution Preparation

In the acute toxicity assay (48 h of exposure), 11 concentrations of the product were evaluated. These concentrations were arranged in a geometric series with a separation factor of 2.2 between successive levels, in accordance with OECD Guideline 202 ^33^ (Table 1). The same concentration range was also used in the hatching rate and hatching speed assays.

**Table 1:** Nominal Concentrations of Active Ingredient Fenthion (mg/L) from the Fenthion-Based Commercial Formulation Thion C Evaluated in the Different Bioassays Conducted with *A. salina*

Type of assay conducted	Tested concentrations (mg/L)
Acute toxicity (48 h)	0.27, 0.60, 1.33, 2.93, 6.44, 14.18, 31.20, 68.65, 151.03, 332.27, 731
Long-term toxicity (14 days)	0.001, 0.002, 0.005, 0.011, 0.024, 0.054, 0.12
Hatching rate and hatching speed	0.27, 0.60, 1.33, 2.93, 6.44, 14.18, 31.20, 68.65, 151.03, 332.27, 731
Inhibition of nauplii growth	0.011, 0.025, 0.056, 0.124, 0.274, 0.604, 1.33

For the long-term toxicity assay, conducted over 14 days, seven concentrations of the product were tested. These concentrations were defined based on a preliminary range-finding test, as presented in Table 1. The concentrations used in the developmental alteration assay, with 72 h of exposure, were established based on the results obtained from the acute toxicity test. In this case, only concentrations that did not cause mortality throughout the entire experimental period were selected. All concentrations tested in the different assays are described in Table 1.

The test solutions were prepared by diluting the commercial product Thion C in artificial seawater to obtain the previously established nominal concentrations (Table 1). The concentrations of fenthion in the test media were not analytically verified during the bioassays. Therefore, all exposure concentrations and effect concentrations (EC_*x*_) derived from the experiments conducted in the present study refer to nominal concentrations, calculated from the fenthion content declared for the commercial formulation.

Artificial seawater was used throughout all experimental procedures, including cyst hatching for nauplii production and the preparation of test solutions used in the different bioassays. The artificial seawater was prepared by dissolving synthetic marine salt (Ocean Tech Reef Active) in previously filtered water to obtain a nominal salinity of 33 ppt. The pH was adjusted to 8.0, in accordance with the conditions recommended by NBR 16530.^34^

In the long term toxicity assay, the artificial seawater was aerated for 48 h before use and was subsequently used to prepare the test solutions. These solutions were always prepared on the day of the assessments and medium renewals to ensure greater stability of experimental conditions throughout the exposure period. For the cyst hatching assay, the artificial seawater was aerated for two consecutive hours before preparation of the solutions and execution of the definitive test.

### Acquisition of *A. salina* Cysts and Nauplii

Cysts of *A. salina*, with a reported hatching rate of 75–80%, were commercially obtained from AquaSchinaider and used as the biological material in ecotoxicological assays to evaluate hatching rate and hatching speed.

For hatching and nauplii production, 0.15 g of cysts were transferred to 1 L of artificial seawater (33 ppt salinity) and incubated for 30 h at 28 ± 2 °C under continuous cool white LED illumination at 300 ± 100 lx. At the end of the incubation period, newly hatched nauplii were collected and used in both short- and long-term exposure experiments, as well as in growth inhibition assays.

### Acute Toxicity Test

Newly hatched *A. salina* nauplii (2 ± 1 h posthatching), maintained in artificial seawater, were used in the acute toxicity assays, conducted in accordance with NBR 16530 guidelines.^34^ The assay was independently conducted on three separate occasions, and the mean immobilization values across these experiments were used for data presentation.

For each tested concentration of fenthion, four experimental replicates were established using sterile 25 mL containers. Ten nauplii were introduced into each experimental unit. Excess water was carefully removed, and 10 mL of the respective test solution (Table 1) was then added. The containers were sealed with plastic film to prevent evaporation and potential variation in experimental conditions.

The assay was conducted under static conditions, without renewal of the test solutions, for 48 h at 24 ± 1 °C under dark conditions in a biochemical oxygen demand (BOD) incubator. Immobilization was assessed every 24 h using a stereomicroscope (Leica). A nauplius was considered immobilized when no movement was observed after gentle agitation of the medium and continuous observation for 15 s. Assay validity required mortality in the negative control not to exceed 10% at the end of the 48-h exposure period, in accordance with NBR 16530.^34^

### Long-Term Toxicity Test

Newly hatched larvae (2 ± 1 h posthatching) showing positive phototactic behavior were carefully collected using a Pasteur pipet, ensuring that no cysts were present in the transferred material. They were then transferred to containers holding 1 L of artificial seawater, where they remained for an additional 24 h at 24 ± 1 °C under continuous cool white LED illumination at 300 ± 100 lx until reaching larval stages II–III, which were considered suitable for the start of the toxicity test.

The definitive test was conducted based on the UNICHIM guidelines^35^ and the protocol described by Manfra et al.,^36^ with adaptations regarding the species used (*A. salina* instead of *Artemia franciscana*), the number of replicates per treatment (six instead of three), the feeding regime (*Spirulina platensis* instead of *Dunaliella tertiolecta*), and the frequency of test-solution renewal. For each fenthion concentration and for the negative control (artificial seawater), six replicates were established. Each replicate consisted of a sterile 100 mL container holding 50 mL of the respective test solution, into which 10 stage II–III nauplii were introduced.

The organisms were fed by adding 250 μL of a *S. platensis* suspension to each container. The suspension was prepared by dissolving 0.5 g of powder in 1,000 mL of artificial seawater, as recommended by Elshafey et al.,^37^ and the powder was obtained from a local compounding pharmacy. After the experimental setup, the containers were sealed with plastic film to minimize evaporative losses. To allow gas exchange, a small hole was made in the center of the film using a sterile syringe.

The experimental units were maintained in a climate-controlled chamber at 25 ± 2 °C under cool white LED illumination at 900 ± 100 lx, with a photoperiod of 14 h light and 10 h dark, throughout the 14-day experimental period. The assay was conducted under semistatic conditions, with renewal of the test solutions every 48 h. Immobilization was assessed under a stereomicroscope (Leica) on days 2, 4, 6, 8, 10, 12, and 14. After each assessment, the nonimmobilized organisms were carefully transferred to fresh test solutions previously supplemented with the food suspension. At the end of the 14-day experimental period, the assay was considered valid only if immobilization in the control group did not exceed 20%.^36^

#### Cyst Hatching Test

The assay was conducted according to the protocol described by Ñañez Pacheco et al.,^38^ with modifications. *A. salina* cysts, with an approximate hatching rate of 75 to 80%, were previously selected for the bioassays. Considering the high intrinsic variability of this biological material, the experiment was performed independently on three separate occasions. Within each independent experiment, a mean value was calculated for each treatment from the five experimental units. Thus, three independent mean values were obtained for each treatment, one from each experiment, and these values were used for statistical analysis and data presentation.

In a preliminary screening step, 0.15 g of cysts were hydrated for 3 h in artificial seawater maintained at 5 ± 2 °C under refrigerated conditions. After this period, floating cysts were discarded, whereas those that settled were considered viable and used in the subsequent steps.

In the definitive test, ten previously hydrated cysts were individually collected using a Pasteur pipet and transferred to containers holding 4 mL of the respective test solutions. For each tested concentration and for the negative control (artificial seawater), five independent containers were used, totaling 50 cysts per treatment.

The test containers were maintained under static conditions, without renewal of the test solutions, at 28 ± 1 °C under continuous cool white LED illumination at 1200 ± 100 lx for 72 h. Evaluations were performed every 12 h under a stereomicroscope, recording the number of free-swimming nauplii as well as individuals at the prehatching, or “umbrella,” stage. A minimum hatching rate of 70% in the negative control after 72 h was adopted as the validity criterion for the assay.

The hatching rate was calculated based on the cumulative number of hatched nauplii relative to the total number of exposed cysts, according to the following expression

1 $$HR\;\left(\%\right)=\left(\frac{\Sigma N}{C}\right)\times 100$$

Where:

HR = hatching rate (%)

∑*N* = total number of nauplii hatched during the experimental period

*C* = total number of exposed cysts

Hatching speed was determined using the hatching speed index (HSI), as follows

2 $$HSI=\frac{N_{1}}{t_{1}}+\frac{N_{2}}{t_{2}}+...+\frac{N_{n}}{t_{n}}$$

Where:

HSI = Hatching speed index

*N_i_* = number of nauplii hatched at time *t_i_.*

*t_i_* = time, in hours, since the beginning of exposure.

### Nauplius Growth Inhibition Test

The experiment was conducted following the protocol described by Shaala et al.^39^ Newly hatched *A. salina* nauplii, with an estimated age of 2 ± 1 h posthatching, were exposed to previously prepared test solutions. For each tested concentration and for the control (artificial seawater), five independent experimental units were established using sterile 25 mL containers. Each unit received 10 nauplii and 10 mL of the corresponding solution, according to the concentrations described in Table 1.

Exposure was static, with no renewal of the test solutions, and was maintained for 72 h in a climate-controlled chamber at 25 ± 1 °C under continuous cool white LED illumination at 900 ± 100 lx. At the end of the experimental period, the 10 organisms from each replicate were carefully recovered, rinsed three times with distilled water, and fixed in 4% formaldehyde solution. Subsequently, specimens were stored at room temperature until morphometric analyses were performed.

To assess development, nauplii were mounted on microscope slides and measured individually under a light microscope (Zeiss) at 40× magnification. Body length (μm) was determined as the distance from the anterior portion of the cephalothorax to the tip of the caudal furca (Figure S1). Images were captured using a 48-megapixel digital camera, and measurements were performed using ImageJ software.

### Data Analysis

For each response variable, the assumptions of residual normality were initially assessed using the Shapiro–Wilk test, and homogeneity of variances was evaluated using Levene’s test implemented in the *car* package.^40^ When the assumptions were met, differences among concentrations were analyzed using one-way analysis of variance (ANOVA) (α = 0.05). When a significant effect was detected, Dunnett’s test (5%), implemented in the *multcomp* package,^41^ was applied to compare each concentration with the corresponding control.

Whenever a concentration–response relationship with a statistically adequate fit was observed, effective concentration values (EC_10_, EC_25_, and/or EC_50_) were estimated through dose–response modeling using the four-parameter log–logistic model (LL.4) implemented in the *drc* package.^42^

All statistical analyses were conducted in R (v. 4.5.2).^43^ Graphs were generated using GraphPad Prism (v. 8.0.1) and the *ggplot2* package.^44^

#### Intraspecies End Point Sensitivity Distribution (iESD) and Species Sensitivity Distribution (SSD)

The iESD was used as a probabilistic approach to integrate variation in sensitivity among the different ecotoxicological end points obtained for *A. salina* exposed to the commercial fenthion-based formulation. Previous applications of approaches based on the distribution of multiple effect measures within a single species have used relatively small data sets, with distributions constructed from 5 to 14 effect measures.^28^ Brain et al.^29^ estimated the first and fifth percentiles using five effect measures, whereas Arts et al.^45^ used at least six EC_50_ values to fit log-normal distributions. Because no universally established minimum number of effect measures has been defined for constructing iESDs, the influence of data set size on lower-percentile estimates was considered by analogy with SSDs, for which smaller data sets are associated with greater uncertainty in HC_5_ estimation.^46,47^

Accordingly, we sought to retain as many effect measures obtained in the present study as possible for fitting the iESD. Previously estimated effect concentrations for the different end points evaluated were used. The EC_10_ was adopted for naupliar growth inhibition because an EC_50_ could not be estimated for this end point, whereas EC_50_ values were used for the other assays. The distribution included immobilization estimates at 24 h, 48 h, 8, 10, 12, and 14 days, as well as estimates for hatching rate, hatching speed, and naupliar growth, totaling nine effect measures. EC_50_ values obtained at different exposure times were retained as specific measures of temporal sensitivity rather than as independent experimental replicates, allowing the iESD to capture changes in sensitivity over the course of exposure. Their inclusion also preserved a larger number of measures for distribution fitting, reducing the dependence of the lower-percentile estimate on a very limited set of observations and contributing to a more stable HC_5_ estimate.

For the SSD construction, EC_*x*_ values obtained for *A. salina* in the present study were combined with fenthion toxicity data retrieved from the literature and ecotoxicological databases (Table S1). Because ecotoxicological data for marine and estuarine organisms were limited, studies using both commercial formulations and the pure active ingredient were included. For each toxicity record, the concentration basis reported in the original source was classified as nominal, analytically measured, or not reported. Of the 20 toxicity records retrieved from the literature, 12 (60%) were based on nominal concentrations, 3 (15%) on analytically measured concentrations, and 5 (25%) did not provide sufficient information to determine the concentration basis (Table S1).

When more than one EC_*x*_ value was available for the same species, the geometric mean was calculated to obtain a single representative value. The same procedure was applied to the data obtained for *A. salina* in the present study, for which the EC_10_ for naupliar growth and the EC_50_ values for immobilization at 24 h, 48 h, 8, 10, 12, and 14 days, hatching rate, and hatching speed were combined by geometric mean to obtain the single species-level value used in the SSD.

The iESD and SSD were fitted in the R environment using the *ssdtools* package,^48^ assuming a log-normal distribution. Model fitting considered concentration values as the lower bound, with automatic data rescaling and restriction to computable distributions, allowing boundary solutions when necessary. Model performance was evaluated based on goodness-of-fit statistics provided by the package.

After model fitting, cumulative distribution curves were generated for both iESD and SSD, representing the potentially affected proportion as a function of concentration. Predictions were then derived across the full fitted curve for proportions ranging from 1% to 99%, at 1% increments, with 95% confidence intervals estimated via nonparametric bootstrap with 5,000 resamples.

From the fitted distributions, the HC_5_ was estimated, defined as the concentration associated with the potential to affect 5% of end points in the iESD or 5% of species in the SSD. The HC_5_ was directly derived from the model, with 95% confidence intervals obtained by nonparametric bootstrap with 5,000 resamples.

Finally, graphical representation of the distributions was constructed by overlaying observed values with the fitted log-normal curve and its corresponding 95% confidence band. The *x*-axis was expressed as log_10_-transformed concentration (mg/L), while the *y*-axis represented the percentage of potentially affected end points or species. The HC_5_ position was highlighted using a dashed line.

### Determination of the Predicted No-Effect Concentration and Risk Quotient

The HC_5_ value obtained from the SSD was used to derive the predicted no-effect concentration (PNEC), which was subsequently employed in the calculation of the risk quotient (RQ), in accordance with the recommendations of European technical guidance documents.^49,50^ The PNEC was estimated by applying an assessment factor (AF), according to the following relationship

3 $$PNEC=\frac{HC_{5}}{AF}$$

In this study, an AF = 5 was adopted, considering that the SSD was constructed using data from at least 10 organisms, consistent with a protective approach for marine and estuarine aquatic ecosystems.^49,50^

The RQ was calculated as the ratio between the measured environmental concentration (MEC) and the PNEC, according to the following expression

4 $$RQ=\frac{MEC}{PNEC}$$

The MEC values for fenthion were obtained from previously published studies,^19,51,52^ considering representative environmental scenarios for estuarine and marine aquatic systems. The different values reported in these studies were used to estimate the minimum, maximum, and mean RQ values. It should be noted, however, that the number of studies focused on the detection and quantification of fenthion in marine and estuarine environments remains limited, which restricts data availability and, consequently, the robustness of MEC estimates.

For the interpretation of the results, a simplified risk classification based on the MEC/PNEC ratio was adopted, considering low risk when RQ < 1 and high risk when RQ ≥ 1.^49,50^

## Results

### Acute Toxicity Test

Mortality in the negative control remained below 10% (Figure 1a), meeting the validity criterion established by NBR 16530. In the acute assay with the fenthion-based commercial formulation, organism immobilization increased as a function of concentration and exposure time.

**Figure 1:**
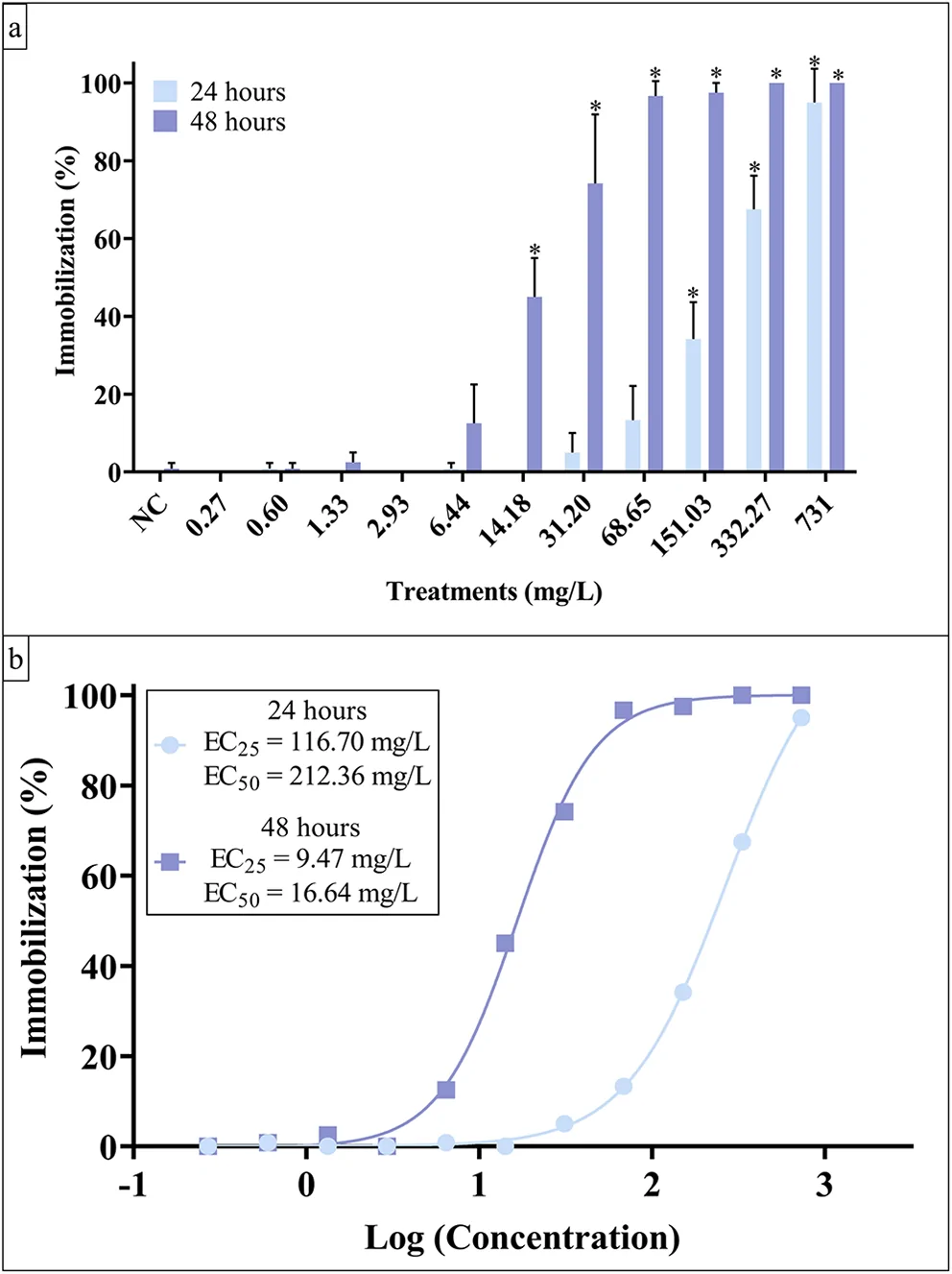
(a) Immobilization rate of *A. salina* nauplii exposed to the commercial formulation based on fenthion for 24 and 48 h. Data are expressed as mean ± standard deviation. The symbol (*) indicates a significant difference compared with the negative control (NC), according to Dunnett’s test, at the 5% significance level. (b) Concentration–response curves for immobilization after 24 and 48 h of exposure, showing the estimated EC_25_ and EC_50_ values.

After 24 h, significant immobilization was observed starting at the concentration of 151.03 mg/L, with 34.16% of organisms immobilized, reaching 95% at 731 mg/L (Figure 1a). The estimated EC_25_ and EC_50_ values for this period were 116.70 mg/L and 212.36 mg/L, respectively (Figure 1b).

After 48 h, significant immobilization was recorded starting at the concentration of 14.18 mg/L, with 45% of organisms immobilized. At the highest concentrations tested (332.27 and 731 mg/L), 100% immobilization was observed (Figure 1a). The estimated EC_25_ and EC_50_ values for 48 h were 9.47 mg/L and 16.64 mg/L, respectively (Figure 1b).

#### Long-Term Toxicity Test

At the end of the experimental period, the mortality observed in the negative control was 13.33%, remaining below the maximum limit of 20% recommended for test validation, thus confirming the acceptability of the results.

For the commercial formulation based on fenthion, after two and 4 days of exposure, no statistically significant effects were observed at any of the tested concentrations. Significant effects began to be recorded from the sixth day of exposure onward. During this period, the concentrations of 0.054 and 0.12 mg/L promoted 18.33% and 35% immobilization, respectively (Figure 2a). The estimated EC_25_ and EC_50_ values were 0.072 and >0.12 mg/L, respectively (Figure 2b).

**Figure 2:**
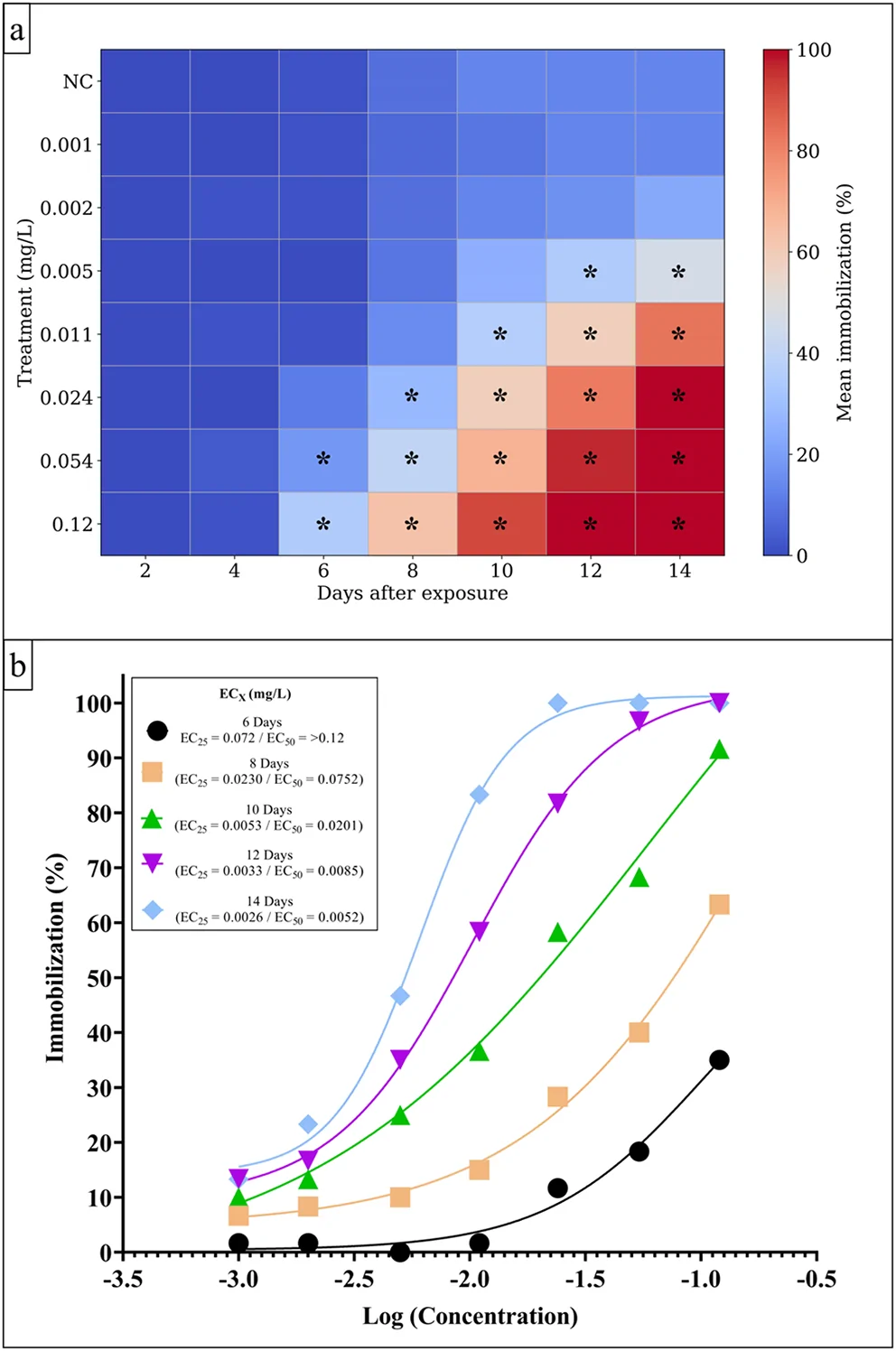
(a) Immobilization of *A. salina* *nauplii* exposed to the commercial formulation based on fenthion for 14 days. Data are presented as a heat map based on mean immobilization (%) as a function of exposure time and the tested concentrations. The symbol (*) indicates a significant difference compared with the negative control (NC), according to Dunnett’s test, at the 5% significance level. (b) Concentration–response curves for immobilization at 6, 8, 10, 12, and 14 days of exposure, with the corresponding estimated EC_25_ and EC_50_ values.

After 8 days of exposure, significant effects were recorded at concentrations of 0.024, 0.054, and 0.12 mg/L, which resulted in 28.33%, 40%, and 63.33% immobilization, respectively (Figure 2a). For this period, the EC_25_ and EC_50_ values were 0.0230 and 0.0752 mg/L, respectively (Figure 2b).

On the tenth day of exposure, significant immobilization was observed starting at the concentration of 0.011 mg/L, which caused 36.66% immobilization. At concentrations of 0.054 and 0.12 mg/L, immobilization rates of 68.33% and 91.66%, respectively, were observed (Figure 2a). The estimated EC_25_ and EC_50_ values were 0.0053 and 0.0201 mg/L, respectively (Figure 2b).

After 12 days of exposure, significant effects were observed starting at 0.005 mg/L, a concentration that resulted in 35% immobilization. At concentrations of 0.054 and 0.12 mg/L, immobilization reached 96.66% and 100%, respectively (Figure 2a). The estimated EC_25_ and EC_50_ values were 0.0033 and 0.0085 mg/L, respectively (Figure 2b).

After 14 days of exposure, statistically significant effects were observed starting at the concentration of 0.005 mg/L, which resulted in 46.66% immobilization. At the concentration of 0.011 mg/L, immobilization increased to 83.33%. At concentrations of 0.024, 0.054, and 0.120 mg/L, total immobilization (100%) was recorded (Figure 2a). For this period, the EC_25_ and EC_50_ values were 0.0026 and 0.0052 mg/L, respectively (Figure 2b).

### Effects on the Hatching of *A. salina* Cysts

The mean hatching rate in the control group after 72 h of exposure was 76.67%, meeting the established validity criterion of at least 70%. Compared with the negative control, treatments at concentrations of 0.27, 0.60, 1.33, 2.93, and 6.44 mg/L showed no significant difference in hatching rate according to Dunnett’s test (*p* > 0.05) (Figure 3a). In contrast, concentrations of 14.18, 31.20, 68.65, 151.03, 332.27, and 731 mg/L significantly inhibited hatching, with reductions of 26.09%, 35.65%, 40.87%, 55.65%, 65.22%, and 75.65%, respectively, relative to the control (Figure 3a). For this end point, the EC_25_ and EC_50_ values after 72 h were 15.97 mg/L and 101.75 mg/L, respectively (Figure 3b).

**Figure 3:**
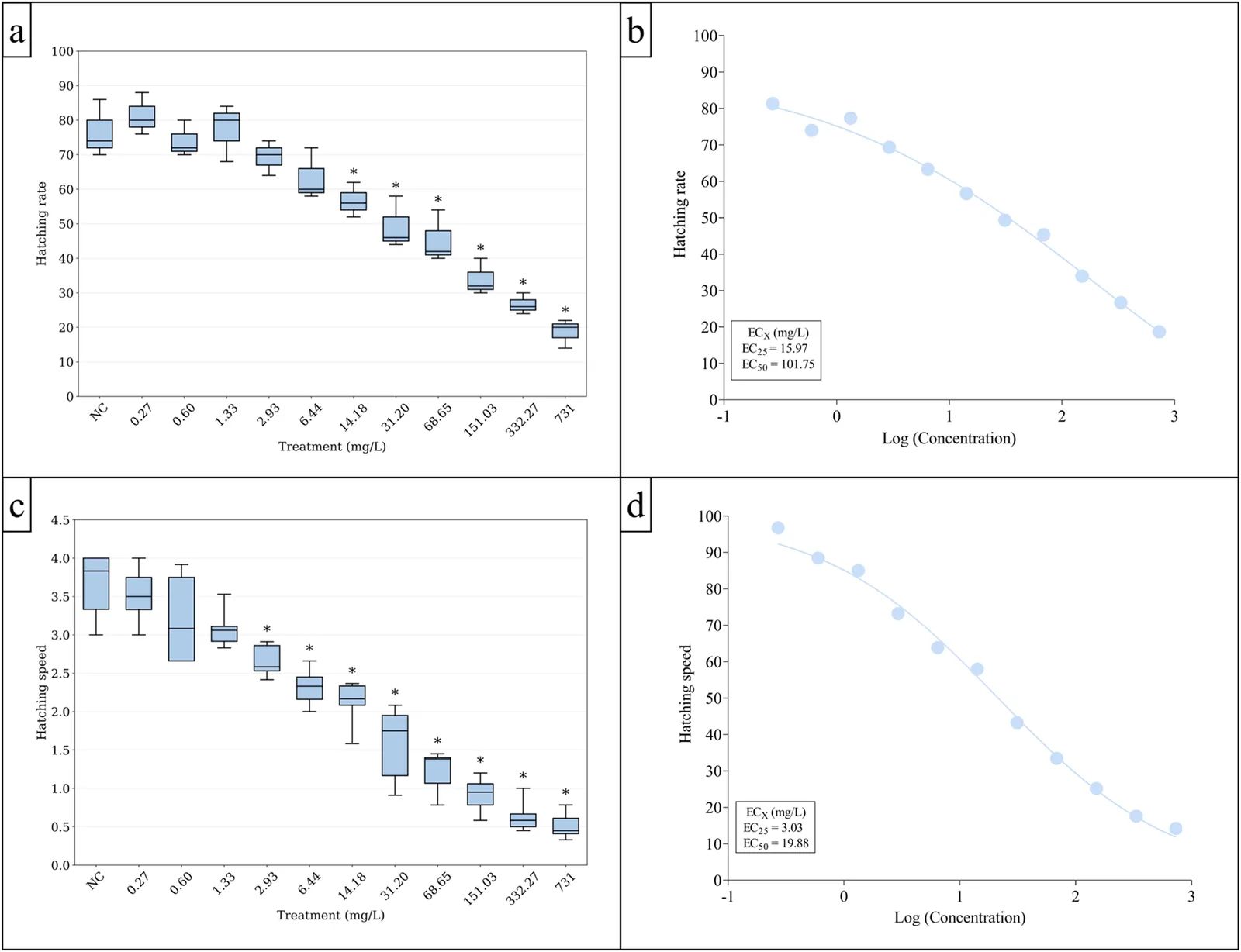
Effects of the commercial fenthion-based formulation on the hatching rate and hatching speed of *A. salina* cysts. In (a, c), boxplots show the distribution of the observed values for hatching rate and hatching speed, respectively, across the tested concentrations, with the central line representing the median. In (b, d), the concentration–response curves fitted to these parameters are shown, together with the corresponding EC_25_ and EC_50_ estimates. The symbol (*) indicates a significant difference from the negative control (NC), according to Dunnett’s test at the 5% significance level.

Regarding hatching speed, treatments at 0.27, 0.60, and 1.33 mg/L also did not differ significantly from the negative control (Figure 3c). As concentration increased, the effects became more pronounced, such that treatments at 2.93, 6.44, 14.18, 31.20, 68.65, 151.03, 332.27, and 731 mg/L resulted in significant reductions of 26.79%, 36.15%, 42.03%, 56.74%, 66.52%, 74.81%, 82.39%, and 85.78% in hatching speed, respectively (Figure 3c). The EC_25_ and EC_50_ values for hatching speed were 3.03 mg/L and 19.88 mg/L, respectively (Figure 3d).

### Effects on Nauplii Development

In the growth inhibition assay, the commercial fenthion-based formulation reduced nauplii development after 72 h of exposure. Compared with the control group, this effect was statistically significant at concentrations of 0.12, 0.27, 0.60, and 1.33 mg/L, with mean reductions of 11.25%, 15.35%, 15.65%, and 16.26%, respectively (Figure 4a). For this end point, the estimated EC_10_ was 0.076 mg/L, whereas the EC_25_ was higher than the highest tested concentration, 1.33 mg/L (Figure 4b).

**Figure 4:**
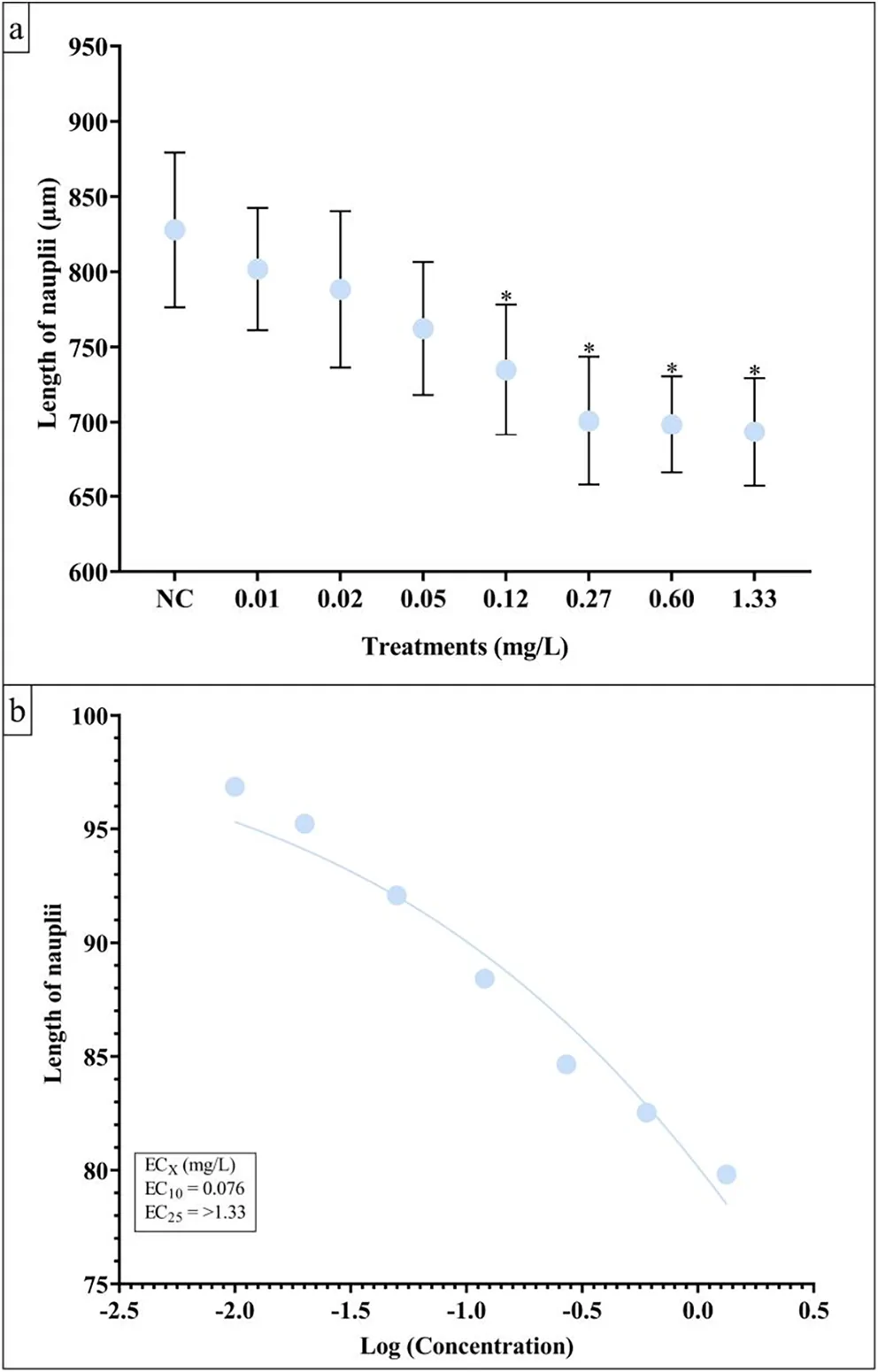
Length (μm) of *A. salina* nauplii exposed to different concentrations of the commercial fenthion-based formulation. In (a), the mean lengths of the nauplii, expressed as mean ± standard deviation, are presented for each treatment. The symbol (*) indicates a significant difference in relation to the negative control (NC), according to Dunnett’s test, at the 5% significance level. In (b), the fitted concentration–response curve for nauplii growth is presented, together with the respective EC_10_ and EC_25_ estimates.

### iESD and SSD

Based exclusively on the values obtained in the present study, the iESD showed that immobilization after 12 and 14 days, in the long term tests, represented the most sensitive end points to the commercial fenthion-based formulation. In contrast, immobilization observed after 24 h and hatching rate represented the least sensitive end points (Figure 5a). The HC_5_ for these end points was estimated at 0.000946 mg/L (CI: 0.000030 to 0.074460 mg/L), equivalent to 0.946 μg/L (CI: 0.030 to 74.460 μg/L) (Figure 5a).

**Figure 5:**
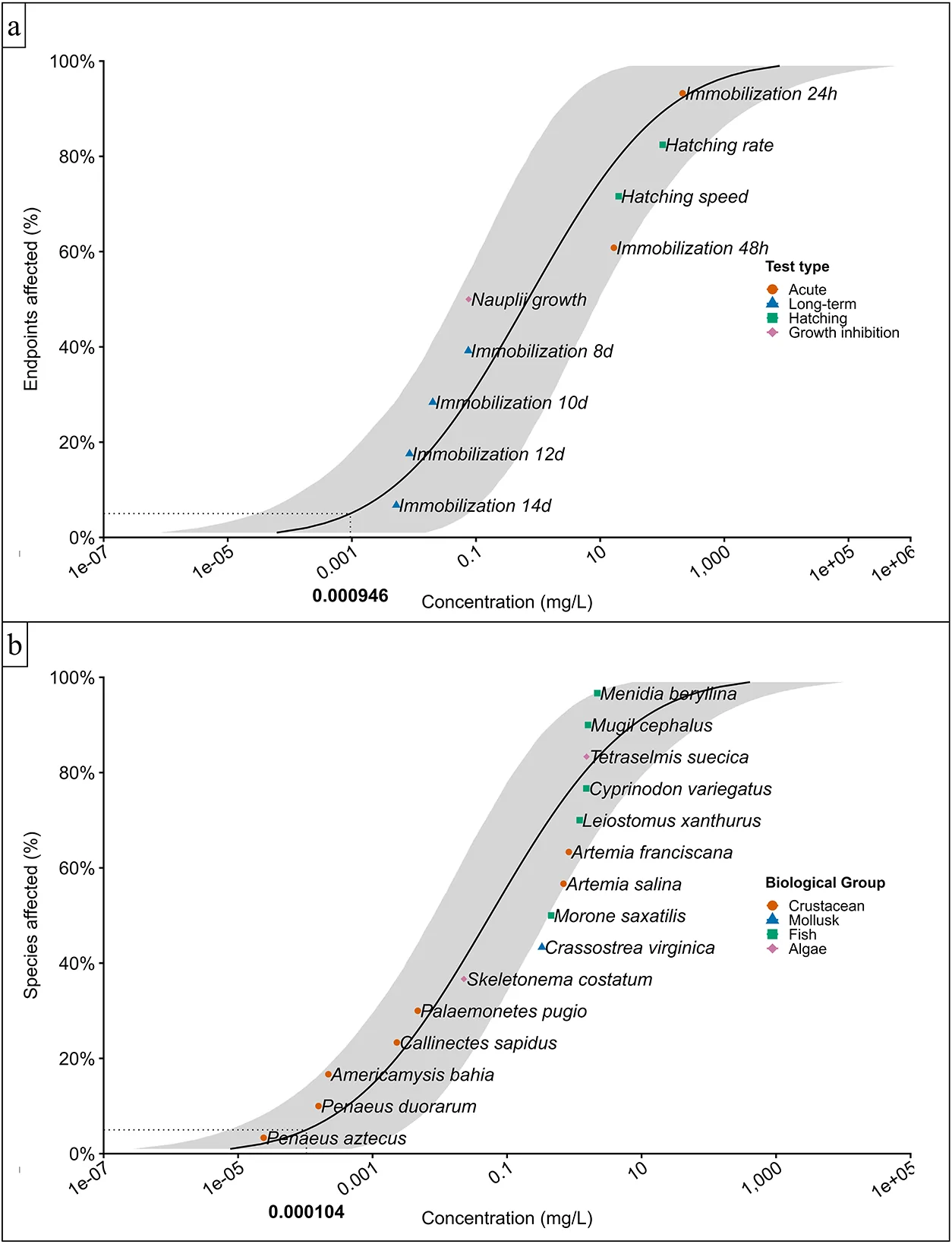
(a) Intraspecies end point sensitivity distribution (iESD) for *A. salina*, constructed using nine effect concentrations obtained in the present study. The values included were: EC_50_ values for immobilization at 24 h (212.36 mg/L), 48 h (16.64 mg/L), 8 days (0.0752 mg/L), 10 days (0.0201 mg/L), 12 days (0.0085 mg/L), and 14 days (0.0052 mg/L); EC_50_ values for hatching rate (101.75 mg/L) and hatching speed (19.88 mg/L); and the EC_10_ for naupliar growth (0.076 mg/L). (b) Species sensitivity distribution (SSD) for fenthion, constructed using ecotoxicity data obtained from the literature (Table S1) and the value derived from the present study for *A. salina* (0.691 mg/L). This value was calculated as the geometric mean of the nine effect concentrations described above in (a). The black line represents the fitted distribution, the gray shaded area indicates the 95% confidence interval of the curve, and the dotted lines indicate the hazardous concentration for 5% (HC_5_) of the end points in the iESD and of the species in the SSD.

Considering the data obtained in the present study together with those reported in the literature for fenthion (Table S1), the SSD showed that the most sensitive species to this compound were *Penaeus aztecus*, *Penaeus duorarum*, and *Americamysis bahia*, whereas the least sensitive were *Menidia beryllina*, *Mugil cephalus*, and *Tetraselmis suecica* (Figure 5b). In terms of community protection, the HC_5_ value was estimated at 0.000104 mg/L (CI: 0.000007 to 0.002763 mg/L), equivalent to 0.104 μg/L (CI: 0.007 to 2.763 μg/L) (Figure 5b).

### Risk Analysis

The estimated PNEC for fenthion was 0.000020 mg/L, equivalent to 0.020 μg/L. Based on the MEC/PNEC ratio, RQ values varied across the evaluated exposure scenarios, with minimum and maximum values of 0.007 and 4.25, respectively. The mean RQ was 1.86 (Figure 6), indicating that, on average, estimated or detected environmental concentrations exceeded the PNEC, suggesting a potential ecological risk to marine and estuarine ecosystems.

**Figure 6:**
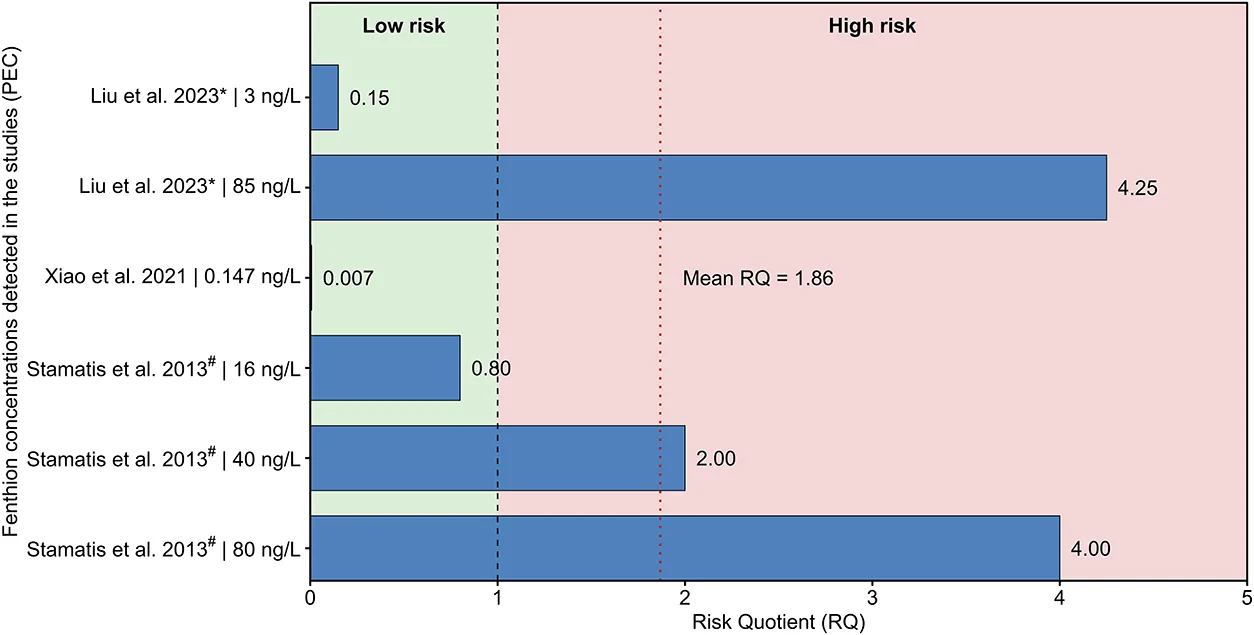
Risk quotient (RQ) values for fenthion based on concentrations reported in the literature for marine and estuarine environments. Each horizontal bar represents an individual concentration derived from the literature and used as the measured environmental concentration (MEC), and RQ values were calculated using the predicted no-effect concentration (PNEC) derived in the present study. Background shading indicates ecological risk categories, with low risk when RQ < 1 and high risk when RQ ≥ 1. *Values reported for fenthion were visually inferred from Figure 2A of the reference study, corresponding to the aqueous phase during the rainy season, as concentrations were neither tabulated nor individually described in the text. The red horizontal line was interpreted as indicative of the mean value, approximately 3 ng/L, while the highest point was considered representative of the maximum observed individual value, approximately 85 ng/L, visually associated with the Pearl River estuary. ^#^The values presented refer exclusively to estuarine zone S6 reported in the study. In this area, 16 ng/L corresponds to the mean fenthion concentration during the period from 2005 to 2007, while 40 and 80 ng/L correspond to the highest values recorded in specific sampling months.

## Discussion

### Effects on the Survival of *A. salina*

The evaluation of pesticides in marine model organisms is essential to reduce uncertainties regarding the effects of these compounds on nontarget biota, especially in scenarios in which they may reach coastal systems through drift, surface runoff, or water transport. In the case of fenthion, the experimental knowledge base is still limited for aquatic organisms, particularly under saline conditions. In this context, by integrating acute and longer-term assays in *A. salina*, as well as the evaluation of sublethal end points, such as hatching and nauplii development, the present study provides quantitative evidence on the sensitivity of the species to the fenthion-based formulation and contributes to the discussion of ecotoxicological risk in marine environments.

The bioassay results indicated that the commercial fenthion-based formulation promoted significant toxic effects in *A. salina* in the acute assays (Figure 1). The EC_50_ estimated after 48 h was 16.64 mg/L (Figure 1b), demonstrating the sensitivity of the species to the tested formulation. To date, the scientific literature reports only one study that evaluated fenthion alone in organisms of the genus *Artemia*. Jaramillo et al.^53^ tested the active ingredient in *A. franciscana* at concentrations of 1, 5, 10, 20, 30, 40, 60, 80, and 100 mg/L and estimated, after 48 h of exposure, an EC_50_ of 0.11 mg/L, a value approximately 151 times lower than that observed in the present study. Additionally, da Silva et al.^5^ evaluated fenthion in association with chlorpyrifos and cypermethrin and reported, after 24 h of exposure, an EC_50_ corresponding to 1.81 mg/L of fenthion in the mixture, in addition to 3.62 mg/L referring to the other compounds.

The variation in EC_50_ values among studies may be related both to the type of product tested and to the species used. In the present study, a commercial formulation was evaluated, whereas Jaramillo et al.^53^ used the isolated active ingredient. The presence of coformulants may modify properties such as solubility, stability, and bioavailability, altering the fraction effectively available to organisms and, consequently, the observed toxicity.^54,55^ In addition, interspecific differences within the genus *Artemia* should be considered, since *A. franciscana* may show greater sensitivity than *A. salina* to certain contaminants, contributing to lower EC_50_ values, as reported by Sarabia et al.^56^ Additionally, comparison with studies that evaluated mixtures, such as that of da Silva et al.,^5^ requires caution, because interactions among compounds may result in additive, synergistic, or antagonistic effects, making EC_50_ estimates not directly comparable to those obtained for isolated compounds or formulations with a single active ingredient.

In addition to the data available for species of the genus *Artemia*, evidence obtained with other marine and estuarine crustaceans indicates high acute toxicity of fenthion in saline environments. The Australian Pesticides and Veterinary Medicines Authority^57^ reported EC_50_ values of 0.000222 mg/L for *Americamysis bahia* (Mysida) after 96 h of exposure, 0.000024 mg/L for *P. aztecus* (Decapoda) after 48 h, and 0.0047 mg/L for *Palaemonetes pugio* (Decapoda) after 48 h. These values are substantially lower than those observed in the present study for *A. salina* (Anostraca) and by Jaramillo et al.^53^ for *A. franciscana* (Anostraca), indicating that sensitivity to fenthion may vary markedly among crustaceans from saline environments. Although such comparisons should be interpreted with caution, given differences in species, exposure time, evaluated end point, and experimental conditions, the available evidence suggests that *A. salina* does not necessarily represent the most sensitive scenario among the marine and estuarine crustaceans evaluated to date, highlighting the need to include other ecologically relevant species in risk assessments for these ecosystems.

In the long term toxicity test, no significant toxic effects were observed during the initial exposure periods of 2 and 4 days. However, toxicity progressively intensified with increasing exposure time (Figure 2a). This pattern is consistent with previous studies showing that the toxicity of fenthion increases with exposure duration in marine and estuarine organisms, although under different exposure regimes,^53,58^ as well as in freshwater organisms.^59^ In the present study, this temporal effect was also reflected in the EC_50_ values (Figures 1b and 2b). In the acute assay, toxicity increased markedly from 24 to 48 h, as indicated by a 12.8-fold reduction in the EC_50_, from 212.36 to 16.64 mg/L. In the long term assay, EC values decreased successively over time, reaching an EC_50_ of 0.0052 mg/L at 14 days. Relative to the value obtained at 48 h, this represents an approximately 3,200-fold decrease, further demonstrating that the toxicity of fenthion increases consistently with prolonged exposure.

To date, no studies have been identified in the scientific literature that have evaluated, over a 14-day period, the effects of prolonged fenthion exposure on the survival of *A. salina*, either as commercial formulations or as the pure active ingredient, highlighting an important knowledge gap regarding the effects of this compound in marine organisms. Nevertheless, evidence obtained for other marine crustaceans supports the plausibility of the effects observed in the present study. In this context, McKenney,^60^ when evaluating the survival of *A. bahia* exposed for 16 days to nominal concentrations ranging from 16 to 300 ng/L, found that 300 ng/L caused marked mortality, reaching 92% on the fourth day and 100% on the ninth day of exposure. Although conducted with a different species, this study supports the present findings by indicating that, under prolonged exposure, fenthion may exhibit high toxicity to marine organisms, even at concentrations in the nanogram per liter range.

From a physiological standpoint, the immobilization observed in both the acute and long-term assays is plausibly associated with the anticholinesterase mode of action of organophosphates, the chemical class to which fenthion belongs. In aquatic invertebrates, including crustaceans, these insecticides inhibit cholinesterases in the nervous system, and cholinesterase inactivation leads to acetylcholine accumulation at synapses, causing continuous stimulation of cholinergic receptors and altered neurotransmission that may culminate in paralysis.^61,62^ This interpretation is also consistent with the relevance of cholinergic pathways to motor regulation in crustaceans^62^ and with evidence from crustacean studies showing that reductions in AChE activity are associated with impaired locomotion and feeding.^63^ Although biomarkers were not measured in this study, this interpretation is consistent with previous evidence showing that organophosphate exposure can inhibit cholinesterase activity in *Artemia nauplii*.^64,65^ In addition, fenthion significantly inhibited cholinesterase activities in *Oreochromis niloticus*,^66^ supporting the biological plausibility that this neurotoxic pathway contributed to the immobilization observed in the present study.

### Effects on Hatching

The commercial fenthion-based formulation promoted moderate effects on the final hatching rate of cysts (Figure 3a) and more pronounced effects on hatching speed (Figure 3c). The difference between the estimated EC_50_ values for hatching rate (101.75 mg/L) and hatching speed (19.88 mg/L) indicates that the latter end point was approximately 5.1 times more sensitive. Because hatching speed reflects the timing of nauplius emergence rather than only the cumulative proportion of hatched organisms at the end of the assay, this lower EC_50_ suggests that the commercial fenthion-based formulation affected hatching dynamics before markedly reducing the final hatching rate. This interpretation is in line with studies on *Artemia* showing that temporally resolved assessments of hatching can provide information on changes in hatching dynamics that may not be fully captured by the final hatching rate alone, including reduced hatching speed, irregular hatching patterns, and gradual inhibition of hatching.^25,38^

Although hatching was recorded at concentrations higher than 14.18 mg/L (Figure 3a), these same concentrations were classified as lethal in the acute bioassay (Figure 1a). In addition, the final hatching rate was less sensitive than the immobilization observed in the acute test, with an approximately 6-fold difference between the EC_50_ values obtained. These results indicate that the emergence of nauplii alone may not adequately represent biological performance, since hatching can still occur at concentrations capable of compromising survival shortly after emergence. This difference in sensitivity has also been reported in the literature, indicating that hatching and posthatching responses may differ in their sensitivity to toxic exposure and that hatching-based assays can provide relevant complementary information when interpreted together with lethality-based parameters.^67–70^

The lower sensitivity of hatching, compared with immobilization, may be explained, at least in part, by the protection conferred by the cyst envelope. Structurally, the *Artemia* cyst presents a multilayered chorion of maternal origin, composed mainly of cortical and alveolar layers, in addition to an underlying embryonic cuticle formed by cuticular membranes and a fibrous layer. This arrangement confers high resistance to hydration and dehydration cycles and acts as a semipermeable barrier, attenuating the penetration of substances present in the medium and reducing direct exposure of the embryo.^71,72^ In toxicological terms, Varó et al.^67^ demonstrated that the chorion hindered the diffusion of the organophosphate chlorpyrifos into the interior of the cyst. In that study, hatching was only slightly affected, whereas postemergence survival was significantly reduced, demonstrating that the cyst barrier may partially buffer embryonic exposure but not prevent the manifestation of toxic effects after hatching.

To date, no studies have been identified that specifically investigated the effects of fenthion on the hatching rate or hatching speed of *A. salina* cysts, nor on dormant embryonic forms of other marine microcrustaceans. This gap is relevant because dormant embryonic forms, such as cysts, resting eggs, or diapause eggs, are not restricted to *A. salina*, but also occur in different groups of marine zooplankton, including copepods and cladocerans, in species such as *Acartia tonsa*,^73^ *Centropages* spp.,^74^ *Labidocera aestiva*,^75^ and *Penilia avirostris*,^76^ in which these structures play a central ecological role in population persistence during environmentally unfavorable periods, by ensuring continuity of the life cycle, and in population reestablishment when environmental conditions once again allow recruitment and development.

In this context, the experimental evidence most directly related to the effects of fenthion on hatching in aquatic organisms refers to embryos of *Danio rerio*, in which exposure initiated at 12 h postfertilization and evaluated up to 72 h, at a concentration of 40 μM, equivalent to approximately 11.13 mg/L, resulted in a significant reduction in larval hatching rate.^77^ This concentration is close to that which also promoted a significant effect on the hatching of *A. salina* in the present study, at 14.18 mg/L (Figure 3a). The authors attributed these effects to the possible inhibition of chorionase, the enzyme responsible for chorion degradation during hatching, as well as to alterations in the embryonic movements required for envelope rupture.^77^

In *Artemia*, hatching also constitutes a physiologically organized process involving cyst hydration, resumption of metabolic activity, envelope rupture, and nauplius release.^78,79^ There is evidence for the participation of enzymes with chorionolytic activity in *A. salina*,^80^ and the final release of the nauplius also depends on its motility within the hatching membrane.^79^ However, although there are structural differences between the chorion of fish and the cyst envelope of *Artemia*, both processes depend on enzymatic activity and coordinated embryonic movements, which supports the plausibility that the fenthion-based formulation interfered with analogous functional stages, as reflected in the observed effects on hatching rate and hatching speed (Figure 3).

From this perspective, the results obtained in the present study with the fenthion-based formulation are consistent with the toxicological pattern described for other organophosphate insecticides in *A. salina*. Rao et al.^81^ reported inhibition of cyst hatchability after exposure to acephate, chlorpyrifos, monocrotophos, and profenofos. Similarly, Baek, Choi, and Rhee^65^ observed reduced hatching in cysts exposed to biocides, including chlorpyrifos and dimethoate. Additionally, Gambardella et al.^25^ reported changes in hatching speed and larval viability following exposure to neurotoxic pesticides such as chlorpyrifos-oxon and diazinon. Thus, although there are no previous studies specifically addressing the effects of fenthion on *A. salina* cysts, the convergence between the findings presented here, the evidence in *D. rerio*, and the data available for other organophosphates in *Artemia* supports the interpretation that the tested formulation interferes with physiological processes essential for hatching, possibly related to enzymatic activity, envelope integrity, and embryonic motility.

### Effects on Naupliar Development

Naupliar growth was sensitive to exposure to the commercial fenthion-based formulation, since significant differences relative to the control were already observed from 0.12 mg/L onward, with an estimated EC_10_ of 0.076 mg/L. This result shows that small changes in larval development could be detected at low concentrations. However, because the EC_25_ was higher than 1.33 mg/L, it can be inferred that higher concentrations would be necessary to cause a more pronounced reduction in growth. Thus, although the formulation was able to alter naupliar development at an early stage, the intensity of the effect on body growth remained relatively moderate under the experimental conditions and over the 72-h exposure period. Considering that, at this early stage, *A. salina* nauplii do not yet depend on exogenous feeding, the reduction in body length likely reflects mainly the direct action of the contaminant on physiological processes involved in larval development, rather than nutritional limitations.^82^

In marine and estuarine organisms, records of growth inhibition caused by fenthion are still scarce, and, to date, no studies have described this effect in *Artemia*. Even so, there is consistent evidence for the estuarine mysid *Americamysis bahia* (syn. *Mysidopsis bahia*). McKenney^60^ exposed the organisms to 16 ± 4, 37 ± 10, 79 ± 18, 166 ± 23, and 300 ± 45 ng/L of fenthion and found that growth was affected in a life stage-dependent manner. The concentration of 166 ng/L significantly reduced the growth rate after 4 days in younger juveniles and in those between 4 and 9 days of age, whereas 79 ng/L delayed the growth of more developed juveniles after approximately 2 weeks. In another crustacean, *Paratya compressa* *improvisa*, exposed to 0.1, 0.2, 0.4, 0.8, and 1.6 mg/L, Hatakeyama and Shiraishi^83^ observed a significant effect only at 1.6 mg/L, with a 40% reduction in body length relative to the control. These findings corroborate the results of the present study with the commercial fenthion-based formulation and indicate that this insecticide may impair the growth of microcrustaceans and other aquatic crustaceans, although the magnitude of the response varies according to species, developmental stage, and exposure concentration.

The inhibitory effects of the fenthion-based formulation observed in *A. salina* nauplii may be associated with the bioenergetic dysregulation described by McKenney and Matthews^84^ in crustaceans exposed to the compound. According to the authors, fenthion increases the metabolic demand for maintenance, thereby reducing the energy available for growth, which is reflected in higher oxygen consumption, lower growth efficiency, and delayed development. Additionally, in vitro studies have demonstrated that fenthion exhibits cytotoxicity^77,85,86^ and the ability to induce DNA damage^77,87^ in different models. Considering that the growth of a living organism depends on adequate cell proliferation and the integrity of DNA replication mechanisms, such alterations may impair physiological processes essential for the growth and development of exposed organisms, such as *A. salina*, thereby corroborating the results observed in the present study.

### iESD, SSD, and HC_5_

The iESD showed that immobilization after 12 and 14 days corresponded to the most sensitive set of end points to the commercial formulation based on fenthion, whereas immobilization after 24 h represented the least sensitive end point (Figure 5a). This variation in sensitivity among end points is biologically plausible, as toxic effects may intensify or become more evident with increasing exposure time. The acute toxicity of organophosphates is classically associated with the inhibition of acetylcholinesterase, the primary mechanism underlying their neurotoxicity.^61,88^ However, under prolonged exposure, the effects of these compounds in microcrustaceans may extend beyond the primary cholinergic target, involving oxidative, metabolic, reproductive, and immune alterations. Although these mechanisms were not directly evaluated in the present study, their potential intensification over time may have contributed to the greater sensitivity of longer-duration end points, positioning them at the lower end of the sensitivity distribution.

The HC_5_ for the commercial formulation based on fenthion estimated by the iESD was 0.946 μg/L, with a confidence interval ranging from 0.030 to 74.460 μg/L (Figure 5a). In comparison, the SSD constructed for fenthion using 15 marine and estuarine organisms yielded an HC_5_ of 0.104 μg/L, with a confidence interval ranging from 0.007 to 2.763 μg/L (Figure 5b). To the best of our knowledge, this is the first study to apply both iESD and SSD within the same ecotoxicological context and to directly compare the values derived from these two distributions. This comparison extends the previously proposed use of lower percentiles from intraspecific distributions, which had mainly been employed as thresholds for hazard and risk characterization.

Brain et al.,^29^ for example, constructed distributions for *M. sibiricum* and *L. gibba* and used the first and fifth percentiles as toxicological thresholds for calculating hazard quotients based on measured or predicted environmental concentrations. These values, however, were not compared with an HC_5_ derived from an interspecific SSD. Consequently, the relationship between the thresholds obtained using the two approaches remained unevaluated. The comparison performed in the present study allows this relationship to be examined, but its interpretation requires consideration that iESDs and SSDs represent distinct sources of biological variation and were developed for different purposes.

An SSD does not provide a directly observed ecotoxicological threshold; rather, it uses effect concentrations obtained from bioassays with different species to statistically characterize the lower tail of interspecific sensitivity.^31,89^ The HC_5_ is therefore a model-derived estimate corresponding to the fifth percentile of the fitted distribution and may serve as a basis for deriving protective values in ecological hazard assessment.^90^ The number of species included influences the uncertainty associated with HC_5_ estimation,^91^ whereas taxonomic coverage and the quality and comparability of the underlying ecotoxicological data influence how well the SSD represents interspecific sensitivity.^30,92^ Accordingly, the HC_5_ should be interpreted not only in terms of its numerical value, but also in relation to the amount, quality, and taxonomic composition of the data supporting the SSD.

The interpretation of a percentile derived from an iESD differs fundamentally from that of an SSD-derived percentile. Rather than estimating the lower tail of sensitivity across species, an iESD-derived percentile is conditional on the set of biological responses evaluated within a single test species.^28,29^ Its main value therefore lies in placing different effect measures on a common probabilistic scale, allowing their relative sensitivity to be compared and comparatively sensitive end points to be identified and prioritized for subsequent assessment. This approach can be particularly useful for organizing and interpreting multiple end points generated for the same species, including situations in which broader interspecific ecotoxicological data are limited.^45^ In this context, the HC_5_ derived from an iESD summarizes the lower end of the distribution of the evaluated responses and should therefore be interpreted in relation to the specific end points from which it was estimated.

The results of the present study reflect this distinction. The HC_5_ derived from the iESD was approximately nine times higher than that obtained from the SSD and, for this data set, its use alone as a protection threshold would result in a less conservative concentration. This difference should not be interpreted solely as a numerical discrepancy between the two methods, because iESD and SSD represent distinct sources of variation in sensitivity and are therefore not interchangeable approaches. For this reason, the iESD was used as a complementary approach to integrate the *A. salina* bioassays, compare end point sensitivity, and characterize intraspecific variation. Environmental hazard characterization, in turn, was based on the HC_5_ derived from the SSD, because this distribution directly incorporates variation in sensitivity among the marine and estuarine species included in the analysis.

Another aspect that should be considered when interpreting the iESD is the species on which it was based. As described in the Introduction, *A. salina* was selected to compose the iESD because of its experimental availability, ecological relevance in saline environments, and the possibility of assessing multiple ecotoxicological end points. However, in the SSD constructed in the present study, *A. salina* was not among the most sensitive crustaceans to fenthion. The species-level toxicity values for *P. aztecus* and *P. duorarum*, for example, were approximately 28,800- and 4,400-fold lower, respectively, than the value used for *A. salina* in the SSD (Figure 5b). This difference in relative sensitivity further supports the interpretation discussed above that values derived from an iESD should not be used in isolation as ecotoxicological thresholds representative of interspecific sensitivity or aquatic communities.

Beyond this species-specific consideration, interpretation of the HC_5_ derived from the iESD should also consider the composition of the distribution itself. In the present study, effect concentrations obtained at different exposure durations were included because toxicity depends on both concentration and exposure time, and effect concentration (EC_*x*_) values may vary over time.^93^ Retaining these estimates allowed the temporal variation in sensitivity observed experimentally in *A. salina* to be represented and avoided a further reduction in the number of entries available for fitting the iESD and estimating its lower percentiles. However, because six of the nine entries corresponded to immobilization assessed at different observation times, with two EC_50_ values derived from the same acute experiment (24 and 48 h) and four from the same long-term experiment (8, 10, 12, and 14 days), the time-specific estimates within each experiment should not be regarded as statistically independent observations. Their inclusion gives greater representation to the immobilization end point and may influence the fitted distribution, the HC_5_ estimate, and its confidence interval. Consequently, the ranking of the effect measures and the HC_5_ should be interpreted in light of this composition.

No studies were found in the current scientific literature that have applied SSD or iESD to fenthion in marine and estuarine organisms. Available estimates are primarily concentrated on freshwater organisms. Nagai,^94^ for example, fitted an acute SSD for fenthion based on data from 31 genera of freshwater arthropods and estimated an HC_5_ of 0.41 μg/L, with a 90% confidence interval between 0.13 and 0.91 μg/L. Similarly, Rizzi et al.,^95^ in a compilation of HC_5_ values for freshwater aquatic species, reported an HC_5_ for fenthion of 1.5 × 10^–3^ μmol/L, equivalent to approximately 0.417 μg/L.

In the present study, the HC_5_ estimated by the SSD using marine and estuarine organisms was 0.104 μg/L, approximately 3.9 times lower than the values reported for freshwater organisms. This difference indicates that the set of marine and estuarine species considered exhibited greater sensitivity to fenthion. Furthermore, it reinforces that values derived from freshwater SSDs may not adequately represent the sensitivity of marine and estuarine organisms, highlighting the need for ecosystem-specific data in the environmental risk assessment of this compound, especially given the scarcity of SSD curves available in the literature for these groups of organisms.

Specifically analyzing the SSD from the present study (Figure 5b), *P. aztecus* is positioned below the HC_5_ value, as the median effective concentration reported for this organism was approximately four times lower than the estimated HC_5_ (Table S1). This result indicates a high sensitivity of *P. aztecus* to fenthion, which is ecologically relevant considering that penaeid shrimp play structuring roles in estuarine and coastal ecosystems.

This ecological relevance becomes even more evident when considering the species’ biology. *P. aztecus* (syn. *Farfantepenaeus aztecus*) has a life cycle closely dependent on connectivity between marine and estuarine environments: spawning occurs in marine areas, whereas the recruitment of postlarvae and juveniles takes place in shallow estuarine habitats, such as marshes and nursery areas, where individuals grow, survive, and later recruit into subadult and adult stages.^96,97^ Thus, the species acts as a biological link between distinct environmental compartments and is particularly vulnerable to contaminants that move across these systems.

From a functional perspective, *P. aztecus* occupies an intermediate trophic position, feeding on plankton during the larval stage and adopting a benthic and omnivorous habit during juvenile and adult stages, consuming worms, algae, microorganisms, and organic detritus.^97^ Therefore, impacts on its survival or population performance may compromise fundamental ecological processes, such as organic matter cycling and energy flow between the benthic compartment and higher trophic levels. In addition, postlarvae, juveniles, and adults are widely consumed by fish, crabs, and other coastal organisms, highlighting their central role in supporting estuarine food webs.^97^ Consequently, population declines of this species have the potential to trigger indirect cascading effects on the structure and functioning of these communities.

From an ecological and conservation perspective, the positioning of *P. aztecus* below the HC_5_ represents a warning signal, as it reveals an inherent conceptual limitation of species sensitivity distributions. Although the HC_5_ is defined based on the protection of 95% of the tested species, this criterion does not explicitly incorporate the functional importance of each species within ecosystems. Therefore, when a species with a disproportionate role in trophic connectivity and nutrient recycling, such as *P. aztecus*, falls within the most sensitive 5%, the threshold derived from the SSD may underestimate the risk of indirect and cascading effects. In such cases, even if most species are statistically protected, the ecological integrity of the system may still be compromised. In this context, a truly protective risk assessment should go beyond the probabilistic framework of SSDs and incorporate functional ecological attributes of the evaluated species, recognizing that species are not ecologically interchangeable and that maintaining ecosystem structure and function may depend on protecting organisms that are both highly sensitive and functionally irreplaceable.

Additionally, it is necessary to consider the methodological limitations of the SSD constructed in the present study. Most of the ecotoxicological data available for fenthion were obtained from acute assays, using end points such as mortality or immobilization over short exposure periods, typically ranging from 24 to 96 h (Table S1). This approach does not capture potential chronic, sublethal, or long-term exposure effects, which may occur at much lower concentrations and affect recruitment, growth, or reproduction. Moreover, the taxonomic composition of the SSD revealed that 46.66% of the included species belong to crustaceans (Figure 5b), introducing a representativeness bias. More critically, there is a complete lack of data for other ecologically relevant groups in marine and estuarine environments, such as polychaete annelids, nonbivalve mollusks, such as gastropods, echinoderms, and elasmobranch fishes, as well as microbial communities and meiofauna. This knowledge gap prevents a more comprehensive assessment of the ecological risk of fenthion in these ecosystems. It is recommended that chronic assays be conducted with species from different functional groups and that the toxicological database be expanded in order to refine future SSD curves and support more realistic and protective environmental quality criteria for this compound.

#### Risk Assessment

The HC_5_ value obtained from the SSD (Figure 5b) was used to derive the PNEC for the insecticide fenthion, resulting in 0.000020 mg/L, equivalent to 0.020 μg/L. The available scientific literature shows a scarcity of explicit aquatic PNEC values for this compound, with only one study directly reporting a numerical estimate. In a risk assessment for freshwater and estuarine environments, Stamatis et al.^51^ estimated a PNEC of 0.0057 μg/L, derived from the most sensitive end point among representative species from different trophic groups (fish, phytoplankton, and zooplankton), applying an assessment factor of 1,000.

Although the values are within the same order of magnitude, the PNEC obtained in the present study was approximately 3.5-fold higher than that reported by Stamatis et al.^51^ This discrepancy is methodologically justifiable. In the present study, the estimate was based on the integration of EC_50_ values from multiple end points and different species distributed across distinct trophic levels. From this data set, the SSD was fitted, from which the HC_5_ was derived, followed by the application of an assessment factor. Thus, the starting point for PNEC derivation does not correspond to a single most sensitive end point, but rather to an integrative probabilistic metric representing the distribution of species sensitivities, incorporating both more and less sensitive responses.

This result indicates that differences among PNEC estimates are primarily associated with the methodological criteria adopted for their derivation, rather than intrinsic variations in fenthion toxicity across studies. Additionally, the influence of biological factors on the observed divergence cannot be ruled out, since physiological and ecological differences between freshwater and marine organisms may affect sensitivity to the compound. In this context, it is noteworthy that the PNEC reported by Stamatis et al.^51^ was derived from freshwater organisms, which may limit its direct comparability with estimates based on marine and estuarine species.

The risk assessment, based on the MEC/PNEC ratio, showed that the insecticide fenthion presented variable RQ values, ranging from low to high depending on the exposure scenario considered. The mean RQ value (1.86) allowed the compound to be classified as posing a high risk to marine and estuarine environments (Figure 6). The interpretation of these results should consider the uncertainty associated with the characterization of the concentrations used in the ecotoxicological data. In the bioassays with *A. salina*, fenthion concentrations in the exposure medium were not analytically determined; therefore, effect concentrations were estimated from nominal values calculated based on the active ingredient content declared for the commercial formulation. The SSD also included literature data based on nominal concentrations, analytically measured concentrations, and, in some cases, records for which insufficient information was available to determine the concentration basis (Table S1). This heterogeneity should be considered when interpreting the HC_5_, PNEC, and the RQs derived from these values.

Although the absence of analytical verification prevents confirmation of the concentrations actually present during the bioassays, experimental data indicate that fenthion is relatively stable in aqueous media. Huang and Mabury^98^ reported hydrolysis half-lives of 59.0 days at pH 7 and 55.5 days at pH 9, at 25 °C. In filtered seawater, Vagi et al.^99^ found that 91.95–93.62% of the nominal concentrations remained after 96 h, even under continuous fluorescent illumination of 4300 lx. These data are relevant because most records included in the SSD were derived from acute tests with exposure periods of 24–96 h, whereas the short-term assays conducted in the present study lasted 48 or 72 h. In the 14-day assay, the organisms were periodically transferred to freshly prepared solutions. Under these conditions, substantial losses due to degradation appear unlikely during short-term exposures comparable to those evaluated in these studies.

These data, however, provide indirect support and do not replace analytical determination of exposure concentrations. Experimental conditions vary among studies, and differences in pH, temperature, light conditions, medium composition, container material, and the presence of coformulants may influence exposure conditions, including the stability, bioavailability, or toxicity of the test substance.^100^ The behavior of the commercial formulation used in the present study may also differ from that of the isolated active ingredient. Therefore, although the available data do not suggest extensive degradation losses during short-term exposures under similar conditions, differences between nominal concentrations and those actually present in the bioassays cannot be excluded.

This uncertainty should also be considered in the risk characterization because the environmental concentrations used to calculate the RQs were analytically measured, whereas the PNEC was derived from an SSD containing ecotoxicological data with different concentration bases. Of the 20 records included in the SSD, only three (15%) were based on measured concentrations (Table S1). Restricting the SSD to these records would substantially reduce taxonomic coverage and representation of interspecific variability. For this reason, the available ecotoxicological data set for marine and estuarine organisms was used, while the concentration the basis of each record was explicitly identified and this heterogeneity was recognized as a source of uncertainty in the derivation of the HC_5_, PNEC, and, consequently, the RQs.

The RQs should therefore be interpreted in light of this uncertainty. An RQ ≥ 1 indicates that the measured environmental concentration exceeds the PNEC derived from the ecotoxicological data set used in this study. However, it is not possible to quantify the extent to which potential differences between nominal concentrations and those actually present in the bioassays affected the HC_5_, PNEC, and the magnitude of the RQs. Even with these limitations, the RQ values and risk classifications obtained in the present study are consistent with the range of risk previously reported for fenthion in assessments conducted in different aquatic systems.

This finding is consistent with the literature, which highlights substantial concern regarding the effects of fenthion on aquatic organisms, particularly invertebrates. In the environmental report of the Australian Pesticides and Veterinary Medicines Authority,^57^ for example, estimated risk quotients for the marine shrimp *P. duorarum* ranged from 4.5 to 88 under runoff scenarios, indicating unacceptable risk for sensitive aquatic invertebrates, whereas for *Daphnia magna*, values ranged from 0.094 to 1.9. This wide variation in RQs, as a function of species and exposure scenario, supports the existence of interspecific differences in sensitivity and reinforces the importance of using multiple test organisms in risk assessment.

Field-based data further support this interpretation. In a monitoring study conducted in the Acheloos River, Greece, which included an estuarine zone, fenthion was among the most frequently detected pesticides (52.6%), and RQ values ranged from 0.18 to 14.75 depending on the year, leading the authors to report high risk to local aquatic ecosystems.^51^ Similarly, Xiao et al.^19^ identified fenthion as one of the main organophosphates in surface waters of the East China Sea, with ecological risk values exceeding 0.1 in all investigated areas and approaching 1, indicating significant environmental concern. Additionally, Li et al.,^52^ investigating the occurrence of the compound in 16 estuarine areas in China, found that in certain locations sampled during the rainy season, fenthion posed a high ecological risk, highlighting the influence of hydrological regimes on exposure and associated risk.

In this context, the results obtained indicate that the insecticide fenthion has a relevant potential to cause adverse effects on marine and estuarine organisms, particularly due to the high sensitivity observed for invertebrates. The risk classification based on the MEC/PNEC ratio, together with the agreement with evidence from field studies, reinforces the consistency and ecological relevance of the present assessment. The inclusion of data obtained with the commercial formulation of fenthion, using *A. salina*, increased the taxonomic representativeness and environmental applicability of the ecotoxicological analyses, contributing to reducing data gaps in estuarine and marine environments. However, it is important to note that SSD-based approaches and risk quotients have inherent limitations, as they do not fully incorporate the complexity of natural ecosystems, such as trophic interactions, sublethal effects, and chronic exposures. Therefore, the findings of this study support the need for complementary and more integrative approaches in the ecological risk assessment of fenthion, particularly in estuarine and marine environments subject to multiple environmental pressures.

## Conclusion

The commercial formulation based on fenthion demonstrated toxicity to *A. salina* across multiple bioassays, with variation in effect intensity among different end points and a progressive increase in sensitivity over the exposure period. In general, acute effects were observed at higher concentrations, whereas prolonged exposures resulted in more pronounced toxicological responses. In addition, effects were detected on ecologically relevant sublethal end points, characterized by slight changes in final hatching rate, reduced hatching speed, and impaired naupliar growth. The iESD developed for the commercial formulation confirmed the pattern of greater sensitivity associated with longer-duration end points, with an intraspecific HC_5_ estimated at 0.000946 mg/L.

The incorporation of data generated with *A. salina* into data sets from other species available in the literature enabled the construction of an SSD and the derivation of reference values for environmental protection in marine and estuarine systems. In this context, a community-level HC_5_ of 0.000104 mg/L and a PNEC of 0.000020 mg/L were estimated. Finally, in the risk assessment, RQs ranged from 0.007 to 4.25, with a mean value of 1.86, indicating a high ecological risk associated with the insecticide fenthion. Overall, these findings highlight the importance of implementing environmental monitoring strategies and adopting more stringent criteria for the assessment and management of fenthion and formulations containing this compound in saline aquatic environments.

## Supplementary Material



## Data Availability

The data supporting the findings of this study are available in the manuscript and its Supporting Information.

## Notes

Representative photomicrograph of an *A. salina* nauplius illustrating the body length measurement procedure used in the growth bioassay (Figure S1); it also includes a compilation of fenthion ecotoxicity data for marine and estuarine organisms obtained from the scientific literature and regulatory databases; together with the results of the present study; the toxicity values, concentration bases (nominal, measured, or not reported), and corresponding data sources used to construct the SSD and estimate the HC_5_ are presented in (Table S1); the Supporting Information also contains the bibliographic references for the studies and regulatory databases consulted to compile (Table S1) (PDF)
