# Supplementary material for: Ecotoxicological Effects of a Commercial Fenthion-Based Formulation on the Survival, Hatching, and Growth of Artemia salina L.: Implications for Risk Assessment in Marine and Estuarine Environments
Source: ACS Omega. 2026 Oct 27;11(44):68679–95. doi: 10.1021/acsomega.6c06726 (PMC13756675; doi:10.1021/acsomega.6c06726)
Supplement: Supplementary file 1 [file ao6c06726_si_001.pdf]

SUPPORTING INFORMATION

Ecotoxicological effects of a commercial fenthion-based formulation on the survival, hatching, and growth of *Artemia salina* L.: implications for risk assessment in marine and estuarine environments

Authors: Leonardo Mendes da Silva[1], Rafael Pereira Ramos[1], Joice Lima Ferreira[1], João Lucas Fonseca Landim[1], Maria Eduarda Magalhães[1], Larissa Fonseca Andrade-Vieira[1*]

Affiliations: [1]Departamento de Ecologia e Conservação, Universidade Federal de Lavras, 37200-900, Lavras, MG, Brazil.

*Corresponding author: Dra. Larissa Fonseca Andrade-Vieira, E-mail: larissa.vieira@ufla.br. Departamento de Ecologia e Conservação, Universidade Federal de Lavras, 37200-900, Lavras, MG, Brazil.

Figure S1. Representative photomicrograph of an *A. salina* nauplius used for body length measurement in the developmental assessment. Body length (red line, µm) was defined as the distance from the anterior portion of the cephalothorax to the tip of the caudal furca.

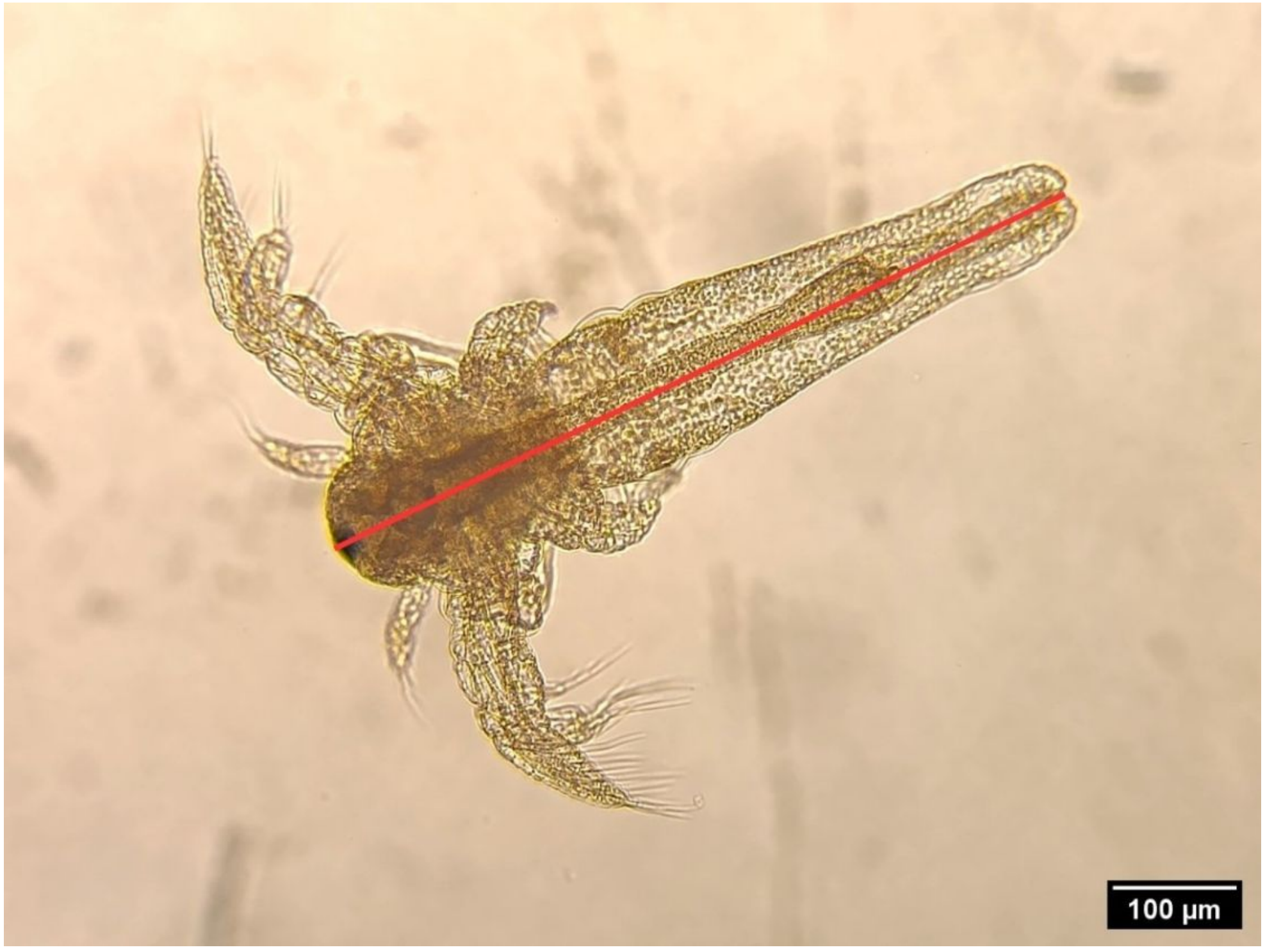

Table S1. Compilation of ecotoxicity data for the insecticide fenthion in estuarine and marine organisms, including values obtained in the present study for *Artemia salina* and data from regulatory databases and scientific studies, used to construct the species sensitivity distribution (SSD) and determine the hazardous concentration for 5% of species (HC5). The concentration basis of each toxicity value is reported as measured, nominal, or not reported.

Organism	Effect concentration(s) (mg/L)	Concentration basis	Reference
Americamysis bahia	0.00022	Measured	101
Penaeus aztecus	0.000024	Nominal	102
Penaeus duorarum	0.00011	Nominal	102
Penaeus duorarum	0.000066	Not reported	102
Penaeus duorarum	24 h = 0.0004; 48 h = 0.00022; 72 h = 0.00015	Not reported	103
Palaemonetes pugio	0.0047	Nominal	102
Callinectes sapidus	0.0023	Measured	102
Crassostrea virginica	0.321	Not reported	104
Crassostrea virginica	0.340	Nominal	102
Cyprinodon variegatus	1.2	Measured	105
Cyprinodon variegatus	1.9	Nominal	102
Menidia beryllina	2.2	Nominal	102
Leiostomus xanthurus	1.2	Nominal	102
Mugil cephalus	1.6	Nominal	102
Morone saxatilis	0.453	Not reported	102

Organism	Effect concentration(s) (mg/L)	Concentration basis	Reference
Skeletonema costatum	0.36	Not reported	106
Skeletonema costatum	0.33	Nominal	102
Skeletonema costatum	0.10	Nominal	107
Tetraselmis suecica	1.52	Nominal	108
Artemia franciscana	24 h = 6.26; 48 h = 0.11	Nominal	109
Artemia salina	Immobilization EC_{50}: 24 h = 212.36; 48 h = 16.64; 8 d = 0.0752; 10 d = 0.0201; 12 d = 0.0085; 14 d = 0.0052<br>Hatching rate EC_{50} = 101.75<br>Hatching speed EC_{50} = 19.88<br>Naupliar growth EC_{10} = 0.076.<br>Geometric mean used in the SSD = 0.691	Nominal	Present study

Note. “Measured” = toxicity endpoint based on analytically determined test-medium concentrations; “Nominal” = endpoint based on prepared concentrations; “Not reported” = concentration basis not clearly specified in the available source.

References

(101) United States Environmental Protection Agency. OPP pesticide ecotoxicity database. Southern IPM Center, **1990**. https://ecotox.ipmcenters.org/details.cfm?recordID=3965 (accessed Apr 16, 2026).

(102) Australian Pesticides and Veterinary Medicines Authority. Environmental chemical review assessment report: fenthion food uses only. Australian Pesticides and Veterinary Medicines Authority: Kingston, ACT, Australia, **2014**.

(103) Clark, G. G.; Weidhaas, D. E.; Norris, L. A.; Levesque, C. S. Field and laboratory toxicity tests with shrimp, mysids, and sheepshead minnows exposed to fenthion. In *Aquatic Toxicology and Hazard Assessment: Ninth Volume*; Mayer, F. L.; Hamelink, J. L., Eds.; ASTM STP 921; American Society for Testing and Materials: Philadelphia, PA, **1986**; pp 123–134. DOI: 10.1520/STP29023S.

(104) United States Environmental Protection Agency. OPP pesticide ecotoxicity database. Southern IPM Center, **1988**. https://ecotox.ipmcenters.org/details.cfm?recordID=9308 (accessed Apr 16, 2026).

(105) United States Environmental Protection Agency. OPP pesticide ecotoxicity database. Southern IPM Center, **1990**. https://ecotox.ipmcenters.org/details.cfm?recordID=3980 (accessed Apr 16, 2026).

(106) United States Environmental Protection Agency. OPP pesticide ecotoxicity database. Southern IPM Center, **1996**. https://ecotox.ipmcenters.org/details.cfm?recordID=9311 (accessed Apr 16, 2026).

(107) Environmental Protection Authority. Fenthion. Chemical classification and information database (CCID). New Zealand Environmental Protection Authority, **2026**. https://www.epa.govt.nz/database-search/chemical-classification-and-information-database-ccid/view/05E66338-C7AC-434E-B2E8-9DC655DCC408/ (accessed Apr 16, 2026).

(108) Vagi, M. C.; Petsas, A. S.; Pavlaki, M. D.; Smaragdaki, N. M.; Kostopoulou, M. N. Toxic effects of the organophosphorus insecticide fenthion on growth and chlorophyll production activity of unicellular marine microalgae *Tetraselmis suecica*: comparison between observed and predicted endpoint toxicity data.

In *Insecticides - Agriculture and Toxicology*; Begum, G., Ed.; IntechOpen: London, **2017**. DOI: 10.5772/intechopen.72321.

(109) Jaramillo, B. E.; Martelo, I.; Duarte, E. Toxicidad aguda de pesticidas organofosforados y análisis de la relación cuantitativa de estructura actividad (QSAR). *Biotecnol. Sect. Agropecu. Agroind.* **2013**, 11, 76–84. https://revistas.unicauca.edu.co/index.php/biotecnologia/article/view/291.
